# Is Tocilizumab Effective and Safe in Polymyalgia Rheumatica and Giant-Cell Arteritis With Polymyalgia Rheumatica?

**DOI:** 10.7759/cureus.27606

**Published:** 2022-08-02

**Authors:** Michelle Farinango, Akhil Ansary, Amulya Dakka, Zahra Nazir, Humaira Shamim, Marie Jean, Muaaz Umair, Pratyusha Muddaloor, Yeny Chavarria, Safeera Khan

**Affiliations:** 1 Internal Medicine, California Institute of Behavioral Neurosciences & Psychology, Fairfield, USA

**Keywords:** giant-cell arteritis with polymyalgia rheumatica, polymyalgia rheumatica, safety, efficacy, il-6 inhibitor, tocilizumab

## Abstract

Polymyalgia rheumatica (PMR) and giant-cell arteritis (GCA) with symptoms of PMR share some pathophysiologic features. Interleukin 6 (IL-6) levels are elevated in both groups. We investigated the effect of tocilizumab (TCZ), an IL-6 inhibitor, in both populations and whether there were any differences regarding effectiveness and safety between them.

We conducted a systematic review following the Preferred Reporting Items for Systematic Reviews and Meta-Analyses guidelines by searching the following databases: PubMed, PMC, Medline, Scopus, Cochrane Library, and ClincalTrials.gov.

We found eight articles including one systematic review, one randomized controlled trial (RCT), one posthoc analysis of an RCT, and five observational studies. A total of 668 patients were included in this study. After a comprehensive analysis, we can only infer that there is insufficient evidence to suggest TCZ as monotherapy. Nevertheless, using TCZ in combination with glucocorticoid can be an effective therapeutic option.

## Introduction and background

Polymyalgia rheumatica (PMR) is a systemic inflammatory condition. The annual incidence ranges from 12 to 60 cases per 100,000 across different populations and increases with age [1]. Moreover, 15-20% of patients with PMR develop giant-cell arteritis (GCA), a large-vessel vasculitis with significant morbidity [2]. Furthermore, it is the second most common inflammatory disease in the elderly after rheumatoid arthritis [3].

PMR frequently affects people over the age of 50. For the majority, it presents acutely. Pain and stiffness in the shoulder and hip girdle are characteristic. However, the neck, upper arms, and thighs are also affected. Low-dose glucocorticoid (GC) therapy is the standard treatment according to the European League Against Rheumatism recommendations and the American College of Rheumatology, mainly because it is effective [1-4]. Nevertheless, long-term treatment is usually needed (>12 months) [1]. Moreover, relapses occurred in 33% of patients while on GC tapering, increasing the time of its use and predisposing patients to developing adverse side effects, such as osteoporosis, diabetes, cataract, glaucoma, infections, and cardio-cerebral vascular events [4,5].

The exact pathogenesis of PMR needs to be elucidated. However, many hypotheses have linked PMR and GCA within the same spectrum of disease. Both pathologies involve the systemic activation of macrophages and T-cells [6]. In addition, high interleukin-6 (IL-6) levels are probably related to the systemic inflammatory response in the two conditions [7,8]. Several studies on PMR have shown a correlation between low circulating IL-6 levels and remission of clinical symptoms. Moreover, high levels of IL-6 after GC treatment are a predictor of relapse [4]. Tocilizumab (TCZ) is a humanized anti-IL-6 receptor antibody. TCZ was approved by the Food and Drug Administration (FDA) for the treatment of GCA with cranial symptoms only, with PMR symptoms only, or both, and at present is being studied as a potential monotherapy or as an adjunctive treatment to GC for isolated PMR [3,9].

Many case reports and observational studies have corroborated the effectiveness of TCZ in isolated PMR and GCA with PMR [5]. Furthermore, in 2020, a systematic review that studied isolated PMR included nine articles: two prospective and seven retrospective studies published between 2012 and 2018 from diverse regions (United States, Europe, and Asia) concluded that TCZ was effective, especially in combination with GC. However, monotherapy was not advocated [4].

This systematic review aims to present new evidence regarding the effectiveness of TCZ as the previous one only included isolated PMR patients and observational studies. Moreover, in clinical practice, physicians encounter patients with PMR only and patients with GCA with PMR symptoms. Because TCZ was approved for the treatment of GCA regardless of the clinical phenotype, we wanted to investigate if TCZ was effective as a monotherapy or in combination with GC, as well as its steroid-sparing effect, in addition to its safety in patients with isolated PMR and GCA presenting with PMR symptoms only.

## Review

Methodology

Protocol

This systematic review followed the Preferred Reporting Items for Systematic Reviews and Meta-Analyses (PRISMA) guidelines [10].

Data Source and Search Strategy

To identify relevant publications, we used the following databases: PubMed, PMC, Medline, Scopus, Cochrane Library, and ClincalTrials.gov. The search was conducted by two different investigators, MF and YC, from February 12 until March 31, 2022. We used the following keywords: Polymyalgia Rheumatica, Giant Cell Arteritis, and Tocilizumab for the PubMed search. In addition, our team used the Medical Subject Headings (MeSH) database to form three different searches in the PubMed search builder using the Booleans OR/AND, as shown in Table 1.

**Table 1:** Three different searches in the PubMed search builder using the Booleans OR/AND.

Keywords	PubMed search builder	Number of articles without filters	Number of articles with filters applied (text availability: full, publication date: 2017–2022)
Polymyalgia rheumatica	(“Polymyalgia Rheumatica/drug therapy”[Majr:NoExp] OR “Polymyalgia Rheumatica/therapy”[Majr:NoExp])	546	111
Giant cell arteritis	(“Giant Cell Arteritis/drug therapy”[Majr:NoExp] OR “Giant Cell Arteritis/therapy”[Majr:NoExp])	926	264
Tocilizumab	(“tocilizumab”[Supplementary Concept]) AND (“Antibodies, Monoclonal, Humanized/adverse effects”[Majr:NoExp] OR “Antibodies, Monoclonal, Humanized/toxicity”[Majr:NoExp])	221	127

Ultimately, we combined the keywords Polymyalgia Rheumatica, Giant Cell Arteritis with Polymyalgia Rheumatica, and Tocilizumab with the Boolean term AND to attain a final search in the PubMed basic search tool, as depicted in Table 2.

**Table 2:** Final search in the PubMed basic search tool.

PubMed search	Number of articles	Number of articles with filters applied (text availability: full, publication date: 2017–2022
Polymyalgia Rheumatica AND Giant Cell Arteritis with Polymyalgia Rheumatica AND Tocilizumab	53	34

Screening Process

Three members of our research team, YC, ZN, and MJ, conducted an independent screening based on our research question, full-text availability, study design, language, and year of publication.

Inclusion and Exclusion Criteria

The citation management software Sciwheel Library collected the articles selected in the screening process, and a tool within the software removed the duplicate papers. Studies underwent a full-text assessment to identify the articles that fulfilled the inclusion and exclusion criteria. Publications considered for inclusion in this study focused on the effectiveness or safety of TCZ in isolated PMR or GCA with PMR symptoms, peer-reviewed, published between 2017 and 2022, written in the English language, and with an experimental, analytical observational, or cross-sectional design. Studies were excluded if they only discussed the effectiveness or safety of TCZ in GCA without PMR symptoms, were non-peer-reviewed or gray literature, were written in languages other than English, and with experimental animal design and descriptive observational studies. Two authors, MC and YC, selected the papers for inclusion, and disagreements were settled by consulting a senior mentor, SK.

Results

**Figure 1:**
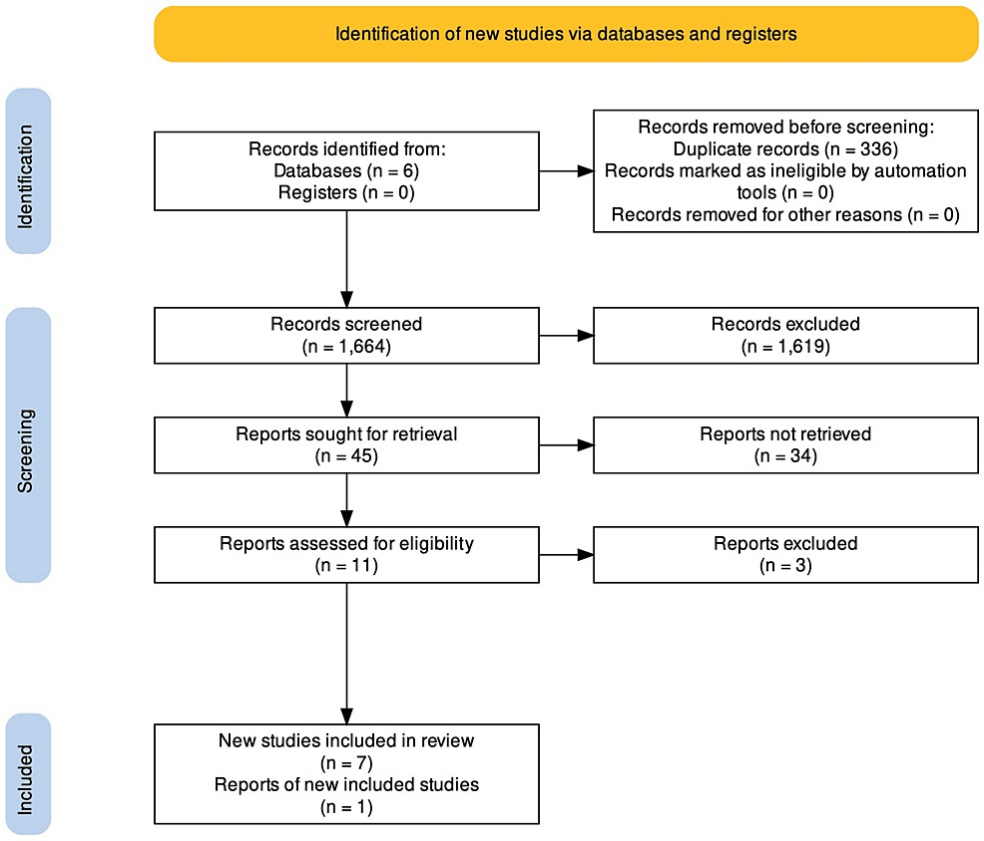
PRISMA flow diagram of the literature search results. PRISMA: Preferred Reporting Items for Systematic Reviews and Meta-Analyses

We identified 2,030 records in our search. After removing the duplicates, 1,664 articles remained for the screening process. Subsequently, 1,619 studies were unrelated to our research topic. Finally, we assessed 11 full-text studies for eligibility and quality appraisal. Three studies were excluded because we did not find data on the statistical significance or the studies did not pass the quality appraisal evaluation. Therefore, we included eight articles in this review [4,11-17]. The PRISMA flowchart is illustrated in Figure 1.

Table 3 summarizes the studies included. We included eight articles, including a systematic review, a randomized clinical trial (RCT), a posthoc analysis of an RCT, and five observational studies. This systematic review consisted of 668 patients in total. We must mention that the systematic review by Akiyama et al. included the TENOR prospective open-label study in which 18 patients participated [4,18]. Additionally, two cohorts and one cross-sectional study included in this systematic review used the data from the participants of the TENOR study to conduct their investigations [13,16,17]. We included these studies in our review as they contained important information regarding additional outcome measures in patients with PMR. We did not add those patients to the final count.

**Table 3:** Summary of the included studies. RCT: randomized clinical trial

Author	Year of publication	Country	Study design	Total of subjects
Bonelli et al. [11]	2021	Austria	RCT	36
Spiera et al. [12]	2021	United States, Switzerland, Canada	Post-hoc analysis of an RCT	250
Akiyama et al. [4]	2020	Japan	Systematic review	59
Carvajal et al. [13]	2017	France	Prospective cohort	36
Izumi et al. [14]	2021	Japan	Retrospective cohort	227
Unizony et al. [15]	2021	United States	Retrospective cohort	60
Huwart et al. [16]	2018	France	Cross-sectional	18
Carvajal et al. [17]	2021	France	Retrospective cohort	36

Usage of TCZ

Two RCTs used 162 mg of subcutaneous (SC) TCZ injections [11,12]. Bonelli et al. applied SC TCZ weekly [11]. Spiera et al. divided the whole group into two subgroups, which received SC TCZ weekly and every two weeks [12].

Five studies implemented intravenous (IV) TCZ, 8 mg/kg, every four weeks [4,13,14,16,17]. One study used SC and IV routes. Information about the dosage and usage was not available [15].

Four studies applied GC concomitantly with TCZ (GC combination) [11,12,14,15], and three used TCZ as monotherapy [11,14,15]. In addition, Akiyama et al. evaluated monotherapy and GC combinations [4], as represented in Table 4.

**Table 4:** Usage of TCZ. PMR: polymyalgia rheumatica; TCZ: tocilizumab; SC: subcutaneous; IV: intravenous; GC combination: tocilizumab plus glucocorticoids; N/A: not available; N/A*: open-label phase, the investigator determined TCZ administration. Data were not available.

Author	Population	TCZ dosage	Administration route	Usage	Monotherapy, GC combination
Bonelli et al. [11]	PMR	162 mg	SC	Weekly	GC combination
Spiera et al. [12]	GCA + PMR	162 mg	SC	Part one: group one: weekly; group 2: every two weeks Part two: group one and two: N/A*	GC combination
Akiyama et al. [4]	PMR	8 mg/kg	IV	Every four weeks	Monotherapy and GC combination
Carvajal et al. [13]	PMR	8 mg/kg	IV	Every four weeks	Monotherapy
Izumi et al. [14]	PMR	8 mg/kg	IV	Every four weeks	GC combination
Unizony et al. [15]	GCA + PMR	N/A	SC and IV	N/A	GC combination
Huwart et al. [16]	PMR	8 mg/kg	IV	Every four weeks	Monotherapy
Carvajal et al. [17]	PMR	8 mg/kg	IV	Every four weeks	Monotherapy

Response to Inflammatory Markers

Two studies investigated the role of IL-6 in patients with isolated PMR. Carvajal et al. demonstrated a strong positive correlation between IL-6 levels with neutrophils (r = 0.767, p = 0.001) and leukocytes (r = 0.715, p = 0.001) in blood [17]. Nevertheless, after TCZ treatment, these associations were not observed. IL-6 also appeared to be strongly linked to disease activity and C-reactive protein (CRP) concentrations (r = 0.7, p = 0.003) [13].

Carvajal et al. also showed an increase in 𝛾 globulins at inclusion and a decrease after TCZ therapy. Additionally, the time for polymyalgia rheumatica activity score (PMR-AS) in the Kaplan-Meier log-rank depicted a significant difference for 𝛾 globulins (p = 0.01). Patients with serum levels of 𝛾 globulins >11 g/dL responded faster compared to patients with ≤11 g/L (hazard ratio (HR) 5.4, confidence interval (CI) = 1.4-20.7) [17].

Another study explored the role of B-lymphocytes in PMR. They found a low absolute peripheral blood CD19+ B-cell count compared to controls (p = 0.04). Regardless of the relative B-cell lymphopenia, total B-cell and switched memory B-cell counts were positively correlated (r = 0.5, p = 0.02), with disease activity assessed by PMR-AS. After the first TCZ dose, only patients with a prompt clinical response (PMR-AS <10 at week four) had notable elevations in absolute B-cell counts [13].

Ultrasound (US) and Magnetic Resonance Imaging (MRI) Changes

Huwart et al. evaluated 28 shoulders and 30 hips with US and MRI in PMR patients treated with TCZ monotherapy. They assessed bursitis at the subacromial-subdeltoid, trochanteric, and iliopsoas sites. Furthermore, glenohumeral and coxofemoral joints were evaluated for bursitis and intraarticular effusion [15]. In general, improvement was found by at least one point in the OMERACT score from baseline to week 12 for US bursitis (p = 0.029), MRI bursitis (p = 0.005), and US effusions (p = 0.001). MRI effusions/synovitis did not change significantly from baseline to week 12 (p = 0.231). MRI was more sensitive to show bursitis improvements than US [16].

Effectiveness of TCZ in PMR and GCA With PMR

The studies analyzed in this systematic review assessed effectiveness by measuring the GC sparing effect, flares, and remission rates.

GC Sparing Effect and Prednisone Discontinuation Rate

In PMR patients, Akiyama et al. reported that two studies showed a >50% reduction of the cumulative GC dose [4]. Bonelli et al. reported a decrease in median cumulative prednisone dose in the TCZ group compared to the placebo group (PBO) at 16 weeks, and it was maintained at 24 weeks (781 mg vs. 1,290 mg) [11]. Izumi et al. evaluated the prednisolone discontinuation rates. Patients were divided by CRP levels at the time of diagnosis. CPR levels <5 mg/dL in TCZ group were associated with 83.3% discontinuation rates and CRP levels >5 mg/dL with 77.9% [13]. In GCA with PMR symptoms, Spiera et al. found a lower cumulative prednisone dose in the TCZ group vs. PBO group (1,862 vs. 3,671.5 mg) [12].

Rate of Flares and Remission Rates

In PMR, Izumi et al. reported no relapses in the TCZ group at the last follow-up visit in their investigation [14]. Bonelli et al.’s primary efficacy endpoint was to reach GC-free remission at week 16. A 63.2% in the TCZ group and 11.8% in the PBO group achieved it (p = 0.002). (odds ratio (OR) = 12.9, 95% CI: 2.2-73.6) in favor of TCZ [11]. Time to first relapse (Kaplan-Meier estimator) favored TCZ (log-rank test; p = 0.007). The estimated mean time to first relapse was 130 days (±13) for the TCZ group and 82 (±11) for the PBO group [11].

In GCA with PMR, Spiera et al. calculated the proportion of patients with more than one flare receiving PBO (57.1%) compared to TCZ (41.9%). Time to first flare was longer in the TCZ group, whereas the risk of flare following clinical remission was similar to PBO (HR = 0.77; 95% CI = O.33-1.78) [12]. Furthermore, Unizony et al. described rates of flares before (75%) and after (34.4 %) treatment with TCZ and a reduction in annual flare rate (p = 0.003). It was also associated with a notable reduction in the time to first flare (HR = 0.2; 95% CI = 0.1-0.5) [15].

Safety

Among patients with PMR, the most common adverse events (AE) were infections, leukopenia, and dyslipidemia [4]. Furthermore, one serious adverse event (SAE), phlegmon, was reported [15].

In GCA with PMR, Spiera et al. mentioned that 96.8% of TCZ-treated patients experienced AE [12]. While Unizony et al. showed that 35.9% of AE were associated with TCZ and 67.9% with either GC or TCZ [15]. The rate of SAE computed by Spiera et al. to be per 100 patient-years (PY) 14.8 (95% CI = 3.1-43.3) for PBO and 28.1 (95% CI = 12.1-55.3) for TCZ [12]. Unizony et al. reported that five patients discontinued TCZ due to an SAE (one event of pneumonia, sepsis, bowel perforation, severe leukopenia, and allergic reaction) [15].

Quality Assessment

MF and YC used the Assessment of Multiple Systematic Reviews tool (AMSTAR 2) depicted in Table 5, the Risk of Bias tool for randomized trials (RoB2), represented in Table 6, and the Joanna Briggs Institute (JBI) for cohort and cross-sectional studies shown in Table 7 and Table 8 to evaluate the methodological quality of the studies and assess the risk of bias in the final articles selected [19-21].

**Table 5:** Quality appraisal tool for systematic reviews. AMSTAR: Assessment of Multiple Systematic Reviews

QA tool for systematic review	Items	Year	Author
AMSTAR 2	11/16	2020	Akiyama et al. [4]

**Table 6:** Quality appraisal tool for randomized clinical trials. RoB: Risk of Bias; RCT: randomized controlled trial

QA tool for RCT	Domains	Risk of biased judgment	Year	Author
RoB 2	5/5	Low	2021	Bonelli et al. [10]
RoB 2	5/5	Low	2021	Spiera et al. [11]

**Table 7:** Quality appraisal tool for cohort studies. JBI: Joanna Briggs Institute

Quality appraisal tool for cohort studies	Items	Year	Author
JBI	8/11	2017	Carvajal et al. [12]
JBI	8/11	2021	Izumi et al. [13]
JBI	8/11	2021	Unizony et al. [14]
JBI	7/11	2021	Carvajal et al. [16]

**Table 8:** Quality appraisal tool for cross-sectional studies. JBI: Joanna Briggs Institute

QA tool for cross-sectional studies	Items	Year	Author
JBI	7/8	2018	Huwart et al. [15]

Discussion

A patient may develop PMR or GCA alone, concomitantly, or at different times in their life. This study exhibits the importance of TCZ as an effective therapy in combination with GC in PMR or GCA with PMR patients. The remission rate falls within >60% using TCZ combined with GC in PMR, while GCA with PMR patients experiences a remission rate of >40%. In addition, a cumulative GC dose reduction of >40% demonstrates the spare effect TCZ has in both populations.

Furthermore, relapse prevention was assessed by a decrease in flares and an increased time to first relapse, again with PMR having a more sustained response than patients with GCA with PMR. Nonetheless, there is still a proportion of patients who experience flares. Currently, one open-label study (NCT01396317) and an RCT SEPHAMORE (NCT02908217) are studying the effects of TCZ with rapid GC tapering in PMR. Additionally, two clinical trials, one open-label (NCT03726749) and a multicenter RCT METOGiA (NCT03892785), are evaluating TCZ with rapid GC tapering and TCZ vs. methotrexate in GCA.

Traditionally CRP and ESR have been used to assess flares. Given that TCZ alters CRP and ESR levels. Practitioners must define a relapse solely on clinical symptoms and imaging, which may be cumbersome. Therefore, research on TCZ should focus on which patients and phases of these diseases should be used [22]. Carvajal et al. concluded that 𝛾 globulins might be utilized to predict a quick response to TCZ therapy [16]. Patients with a serum level of 𝛾 globulins >11 g/dL responded faster than patients with ≤11 g/L in patients with PMR. This could prompt which patients should initiate TCZ in combination with low vs. high GC dose. Therefore, further investigations should be done on PMR and GCA with PMR patients.

According to different data, B cells may play a role in the immunopathology of PMR and GCA [23]. PMR activity was linked to peripheral B-cell lymphopenia, abnormal B-cell distribution, and increased levels of IL-6. However, Carvajal et al. reported that TCZ monotherapy corrected these changes [13]. Moreover, US bursitis, MRI bursitis, and US effusions improved by at least one point in the OMERACT score [16]. Akiyama et al. concluded in their systematic review that TCZ monotherapy was associated with a slow response and may not be as effective as GC in suppressing disease activity [4]. However, the authors employed three studies with a small sample to deduct it. Currently, no clinical trials are studying TCZ as monotherapy. Hence, the evidence supporting it as a single agent is insufficient.

The role of TCZ in patients with GCA is better defined. In 2017, the FDA approved it, following the phase 3 Giant-Cell Arteritis Actemra (GiACTA) trial [9]. A posthoc analysis of this trial showed the effectivity of TCZ with GC tapering regardless of the clinical phenotypes in GCA [12]. These findings align with a retrospective analysis by Unizony et al. They concluded that PMR in patients with GCA did not impact the response to TCZ [15].

We review the different doses and forms of administration that TCZ was given. Two RCTs used 162 mg SC weekly, one systematic review, and five observational studies used 8 mg/kg IV every four weeks. Finally, one retrospective single-center analysis used SC and IV forms [4,11-17]. Regardless of the route and dose used, TCZ was effective. Yet, SC TCZ generated a lower response in remission and cumulative GC dose reduction than IV TCZ. Different study designs, patient characteristics, and GC combination scheme strategies may have contributed to the difference in response rate. Mainly TCZ was safe. In patients with PMR, Bonelli et al. found a total number of AE per 100 PY of 490.6 (95% CI = 468.9-523.2) [11]. In contrast in GCA with PMR, Spiera et al. disclosed a total number of AE per 100 YP of 1,049.6 (95% CI = 934.0-1,175.6) [12]. In all of the studies, infections were the most common AE. SAE occurred in a scant number of patients, serious infections being the most common [4,11-17]. Consequently, it is prudent to be precautious when prescribing TCZ to patients with risk factors for developing infections.

This systematic review has diverse limitations. Because some authors consider PMR and GCA with PMR to be part of the same disease, we studied these two types of populations as it was unclear whether TCZ was effective, and we wanted to explore any variations that may exist between the two of them. Nevertheless, patients with PMR were more than GCA with PMR making our sample unbalanced. In addition, unpublished and gray literature articles were omitted, as well as papers written in languages other than English due to a lack of translational resources.

## Conclusions

Our systematic review highlights the importance of the clinical phenotypes of PMR and aims to answer the question of whether TCZ is effective and safe. We can only infer that there is insufficient evidence to suggest its use as monotherapy. Nevertheless, using TCZ in combination with GC can be an effective therapeutic option because it decreases the rate of flares, increases the time to first relapse, and reduces the cumulative GC dose. Regarding its safety, most adverse events were related to infections, but overall, TCZ was considered safe. However, a definitive conclusion cannot be made because a meta-analysis was not performed. Hence, new research investigations should be done by performing a meta-analysis to confirm this.
